# The Multifaceted Role of Macrophages in Oncolytic Virotherapy

**DOI:** 10.3390/v13081570

**Published:** 2021-08-09

**Authors:** Laura Hofman, Sean E. Lawler, Martine L. M. Lamfers

**Affiliations:** 1 Department of Neurosurgery, Brain Tumor Center, Erasmus Medical Center, Wytemaweg 80, 3015 CN Rotterdam, The Netherlands; l.hofman@students.uu.nl; 2 Department of Neurosurgery, Brigham and Women’s Hospital, Harvard Medical School, 75 Francis St., Boston, MA 02115, USA; slawler@bwh.harvard.edu

**Keywords:** cancer immunotherapy, oncolytic virotherapy, tumour microenvironment, innate immune system, tumour-associated macrophages, M1/M2 phenotypic shift, anti-tumour immunity

## Abstract

One of the cancer hallmarks is immune evasion mediated by the tumour microenvironment (TME). Oncolytic virotherapy is a form of immunotherapy based on the application of oncolytic viruses (OVs) that selectively replicate in and induce the death of tumour cells. Virotherapy confers reciprocal interaction with the host’s immune system. The aim of this review is to explore the role of macrophage-mediated responses in oncolytic virotherapy efficacy. The approach was to study current scientific literature in this field in order to give a comprehensive overview of the interactions of OVs and macrophages and their effects on the TME. The innate immune system has a central influence on the TME; tumour-associated macrophages (TAMs) generally have immunosuppressive, tumour-supportive properties. In the context of oncolytic virotherapy, macrophages were initially thought to predominantly contribute to anti-viral responses, impeding viral spread. However, macrophages have now also been found to mediate transport of OV particles and, after TME infiltration, to be subjected to a phenotypic shift that renders them pro-inflammatory and tumour-suppressive. These TAMs can present tumour antigens leading to a systemic, durable, adaptive anti-tumour immune response. After phagocytosis, they can recirculate carrying tissue-derived proteins, which potentially enables the monitoring of OV replication in the TME. Their role in therapeutic efficacy is therefore multifaceted, but based on research applying relevant, immunocompetent tumour models, macrophages are considered to have a central function in anti-cancer activity. These novel insights hold important clinical implications. When optimised, oncolytic virotherapy, mediating multifactorial inhibition of cancer immune evasion, could contribute to improved patient survival.

## 1. Introduction

Increasing research into the role of the immune system in tumour formation and progression has revealed immune cell evasion to be a central cancer hallmark [1]. This has led to the development of cancer immunotherapies designed to counteract the immunosuppressive characteristics of the tumour microenvironment (TME) and to target tumour antigens. Oncolytic virotherapy is a form of immunotherapy and refers to the administration of viruses that conditionally replicate in tumour cells and activate the immune system [2]. However, the type of immune stimulation induced by oncolytic viruses (OVs) may affect the therapeutic outcome, either by inhibiting the establishment of viral infection or by inducing an anti-tumour immune response [3]. The aim of this review is to explore and elucidate the role of macrophage-mediated responses in the interaction with OVs and the efficacy of oncolytic virotherapy. Understanding the host response is clinically relevant, rendering clues for novel strategies to potentially optimise therapeutic outcomes.

## 2. Tumour Microenvironment (TME)

Molecular alterations driving cancer growth affect not only the tumour cell but also cells in its environment. Tumour cells have evolved multiple strategies for immune evasion, including disruption of interferon (IFN) signalling [4,5] and secretion of immunosuppressive factors [6]. Other cells in the TME subsequently acquire evasive immune programmes. These heterologous cell types can actively affect the clinical response to treatments, thus constituting an important determinant in therapeutic outcome [7].

### 2.1. Innate Immunity in the TME

In the TME, macrophages play a central role in regulating other components of the immune system and are present at all stages of tumour progression [8]. Most tumour-infiltrating macrophages are derived from blood monocytes, that continuously renew non-proliferating local macrophages [9,10]. The presence of chemokine C-C motif ligand 2 (CCL-2), or monocyte chemoattractant protein 1 (MCP-1), in the TME, produced by both tumour cells and stromal cells, has been shown to attract macrophages and subsequently maintain chemotaxis by stimulating these macrophages to also secrete CCL-2 [11]. Clinical data suggest that high macrophage density within the TME correlates with a poor prognosis [12] and poor response to treatment [13,14,15].

Tumour-associated macrophages (TAMs) are among the most prevalent tumour-infiltrating immune cells, comprising up to 50% of the tumour mass [12,16]. In order to exert their multiple functions, macrophages can adopt divergent phenotypes with their polarisation states, which can be broadly termed M1 and M2. This dichotomy, however, simplifies a spectrum of intermediate phenotypes that macrophages acquire through continuous and dynamic microenvironmental signalling [17]. The classically activated M1 phenotype primarily has inflammatory properties and exhibits active signalling involving signal transducer and activator of transcription 1 (STAT1), interferon regulatory factor 5 (IRF5), and nuclear factor κ-light-chain enhancer of activated B-cells (NF-κB). This results in the upregulation of major histocompatibility complex class II (MHC-II) and co-stimulatory molecules [18,19]. M1 macrophages recruit and activate other inflammatory immune cells such as natural killer (NK) cells and dendritic cells (DCs) [20]. Through phagocytosis, M1 macrophages can mediate the (cross-)presentation of antigens to elicit an adaptive immune response. M1-like macrophages are considered tumour-suppressive since they can induce tumour cell death directly through macrophage-mediated cytotoxicity or indirectly by instigating an immune response against tumour-associated antigens (TAAs). The alternatively activated M2 phenotype exhibits active STAT6-IRF4 signalling [18] and exerts wound healing and repair functions [21]. This is associated with the secretion of extracellular matrix remodelling molecules and immunosuppressive cytokines, such as interleukin 10 (IL-10) and transforming growth factor β (TGF-β) [20]. These macrophages enable efferocytosis of apoptotic cells, clearing them without inducing an immune response [22]. M2 macrophages also express proliferation-stimulating [23] and pro-angiogenic [10] signals, features that support tumour growth and metastasis [24,25,26,27,28,29].

In vivo, the diversity of TAMs is not yet fully explored [30]. Many tumours have upregulated expression of CD47, a cell surface molecule that interferes with phagocytosis. This indicates that phagocytic processes, associated with the presence of M1-like macrophages, could be active in the TME, however, they are counteracted by immune evasive strategies developed through selection pressure [31]. These findings illustrate the intrinsic functional plasticity of macrophages and their potential to be converted into other subtypes in response to changing environmental conditions [32]. The phagocytic activity of M1 macrophages contributes to their antigen-presenting cell (APC) properties, which gives them a bridging function linking innate and adaptive immune systems [17].

### 2.2. TAMs Affect Adaptive Immunity in the TME

TAMs can communicate with the adaptive immune system through the production of immunosuppressive cytokines, including chemokines that recruit regulatory T-cells (T_REG_s). TAMs and T_REG_s further contribute to local adaptive immunosuppression by secreting IL-10, which functionally impairs infiltrating T-cells by interfering with their production of pro-inflammatory IFN-γ [33,34]. In conjunction with IL-10 signalling, T-cell expression of negative regulators of T-cell activation such as programmed cell death protein 1 (PD-1) is induced [35]. It has been demonstrated that the depletion of TAMs, but not of normal tissue macrophages, restores tumour-infiltrating cytotoxic T-lymphocyte (CTL) responses and suppresses tumour progression [9].

In summary, cancer immune evasion is initiated by molecular aberrations in tumour signalling pathways, which influences local innate and adaptive immune cells. TAMs play a central role in immune regulation and can adopt divergent phenotypes depending upon their polarisation status acquired through microenvironmental signalling. Generally, they support tumour growth through their immunosuppressive properties.

## 3. Oncolytic Virotherapy

The suppressive characteristics of the TME have provided a rationale for targeting the immune system, which has led to immunomodulation as a therapeutic strategy. OV-based immunotherapies are showing great promise for various cancer types, with the first OVs making it to registration or receiving fast-track designation by the U.S. Food and Drug Administration (FDA) [36]. Both naturally tumour-selective or oncotropic as well as engineered OVs are under investigation. The effects of OV application are a reflection of the biology of natural viruses and their (co-)evolved interactions with the host and its immune system [37]. OVs, therefore, represent a class of therapeutic agents that can affect anti-cancer activity through a dual mechanism of action of cell lysis and immune stimulation.

### 3.1. Oncolysis and Immunogenic Cell Death (ICD)

Tumour cell lysis was long considered the predominant therapeutic mechanism of OVs. In this setting, constitutive viral replication would be required until each tumour cell has been eliminated. It was widely assumed that the host’s immune system limits the clinical efficacy of oncolytic virotherapy by prematurely eradicating the viruses [38]. Now, the importance of the immune system in its anti-cancer activity is well-recognised [39]. Anti-viral responses and anti-tumour responses were both found to be induced by OVs. These immune responses were shown to improve overall therapeutic outcome [40]; survival advantages seen in immunocompetent tumour models were lost in immunodeficient and immunosuppressed models [41,42,43]. The central aim of OV research has, therefore, shifted from the induction of oncolysis to the induction and stimulation of anti-tumour immune responses.

Although the activity exerted by OVs extends beyond the infection of individual tumour cells, some degree of viral replication and oncolysis is required to establish subsequent immune activation. Moreover, anti-cancer activities are connected by the propensity of many OVs to induce immunogenic cell death (ICD), which is characterised by the exposure of damage- or pathogen-associated molecular patterns (DAMPs and PAMPs), and the release of cytokines, including type I IFNs and IFN-γ [44,45,46,47]. In addition to these danger signals, infected tumour cells will release OV progeny and TAAs, together acting as potent activators of the immune system.

### 3.2. Immune-Mediated Mechanisms

The immune responses that OVs induce are two-sided; therapeutic effects are dampened by the immune system naturally responding to deactivate the virus and promoted by an anti-tumour response. OV replication within the TME confers a three-way interaction between tumour cells, OVs, and the immune system establishing a critical balance that determines the therapeutic outcome.

An immediate, humoral host response against the applied OV is mediated by complement activation and pre-existing antibodies against prevalent human viruses—or their induction through multiple administrations. This could result in neutralisation, opsonisation, and rapid clearance of free OV particles and infected cells [48]. Intrinsic, intracellular defence is induced through pattern recognition receptors (PRRs), such as Toll-like receptors (TLRs), that are activated upon PAMP recognition [49]. TLRs signal via the myeloid differentiation primary response protein 88 (MYD88), which leads to activation of the transcription factor NF-κΒ inducing upregulated expression of MHC-I [50] and transcription of pro-inflammatory cytokines [51]. Virus infection leads to activation of innate immune cells such as macrophages, DCs and NK cells [52]. Both inflammation and cellular innate immune mediators in the TME form an extracellular anti-viral barrier, in addition to the cancer type-dependent density of the extracellular matrix that physically contributes to the restriction of viral spread [53]. These activated cells affect the lysis of OV-infected cancer cells as well as provide a link to adaptive immune cells that are recruited by the inflammatory TME and that react against virus-specific antigens [54]. These responses directed against the virus may also directly contribute to the anti-cancer activity. It is now proposed that innate defence, through secretion of pro-inflammatory cytokines, may have cytotoxic effects on other uninfected tumour cells [55,56,57]. Moreover, a correlation between anti-viral and anti-tumour T-cell responses suggests ‘epitope spreading’, where a strong response against a viral antigen leads to effects against other antigens nearby, indirectly eliciting an anti-tumour response [58].

Anti-viral responses generally convert immunologic tolerance to active inflammation in the TME and hence enable the induction of an anti-tumour response. As viral progeny and TAAs are released into the TME and circulation, these antigens can be taken up and processed by APCs, leading to antigen presentation to T- and B-cells. This may elicit an adaptive response against virus-specific antigens as well as against tumour cells expressing TAAs. Administration of OVs has resulted in tumour regression and disease control in injected and distant non-injected tumours [47,59,60,61,62]. Such abscopal effects are attributed to a systemic immune response that has been shown to be T-cell-mediated [41,59,62,63,64,65,66]. Accordingly, OVs are considered in situ vaccines.

Thus, the suppressive TME allows for immunomodulation as a therapeutic strategy. For oncolytic virotherapy, anti-cancer activity was initially thought to consist predominantly through direct oncolytic cell death, rendering anti-viral immune responses an impediment. With the application of immunocompetent animal models, it was discovered that OV-induced ICD elicits inflammation, affecting the suppressive TME, which ultimately enables innate immune cell cytotoxicity and the induction of adaptive T-cell-mediated anti-tumour responses.

## 4. OV-Induced Macrophage-Mediated Responses

The balance between anti-viral and anti-tumour immune responses ultimately determines the anti-cancer efficacy of oncolytic virotherapy. A role in initial anti-viral responses is mainly attributed to OV-induced innate immunity. The reciprocal impact of OVs on macrophages is dynamic, rendering the role of macrophage-mediated responses in the efficacy of oncolytic virotherapy under investigation.

### 4.1. Obstructive Macrophage Responses

In early response to oncolytic virotherapy, macrophages can have a barrier function that limits the delivery and spread of OVs from the site of administration to the tumour and within the TME. Rapidly responding to PAMPs, systemic monocytes and macrophages with an inflammatory M1 phenotype may mediate an anti-viral reaction [19]. Receptor-mediated, accelerated clearance is elicited by complement proteins and antibodies opsonising OV particles, blocking their ability to interact with cellular receptors and facilitating their recognition by macrophages [67]. Within the tissue, macrophages exhibit anti-viral activity through the production of type I IFNs, as was substantiated by the observation that local depletion reduces type I IFN production [68]. Phagocytosis of OVs results directly in the clearance of OV particles [69] and of infected tumour cells, or indirectly through viral antigen presentation and induction of adaptive immunity. In glioblastoma multiforme (GBM) mouse models, M1 microglia (brain-resident macrophages) have been found to clear oncolytic vaccinia virus (VV) particles by immediate uptake after intratumoural delivery [70]. Together, these results led to the notion that enhanced therapeutic efficacy may require specific suppression or depletion of these innate immune cells – extending upon the trend of generally restraining the immune system in effectuating anti-viral immune responses. The use of innate immunomodulatory agents, or the use of OVs as vectors encoding proteins that directly interfere with anti-viral defence molecules, has been shown to increase OV replication, spread, and direct oncolysis [71].

A first immunosuppressive agent investigated in conjunction with OVs for general suppression of the innate immune system is cyclophosphamide (CPA), which is, notably, also an approved anti-cancer chemotherapeutic. Co-administration was shown to allow for OV dose reduction [72] and increased the number of infected cancer cells and viral replication for several OVs in different tumour models [73,74]. CPA pre-treatment has been found to increase viral propagation, oncolysis and therapeutic efficacy, suppressing mRNAs of several anti-viral cytokines such as IFN-α/-β, -γ and TNF-α in peripheral blood mononuclear cells [75], intratumoural phagocyte infiltration and IFN-γ production [76], as well as B-cell responses [77,78], while increasing CTL infiltrates and inducing type 1 helper T (T_H_1-)cell immunity on a systemic level [79]. Further, it has been demonstrated to specifically mediate ablation of the TAM population [80]. The application of CPA in combination with complement inhibitor cobra venom factor, suppressing both cellular and humoral innate responses, effectuated enhanced viral infection and propagation of an oncolytic herpes simplex virus (HSV-)1 within tumours in vivo and consequently increased the life span of GBM-bearing rats [81]. TGF-β treatment, known to inhibit NK cells, inflammatory macrophages, and microglia, resulted in enhanced oncolytic HSV-1 titers and suppression of tumour growth in syngeneic murine GBM models [82].

A similar increase in OV titers in the TME could be achieved by direct depletion of macrophages and microglia. This depletion has been investigated as a strategy to enhance the systemic delivery of oncolytic virions [83]. A commonly used approach consists of the application of clodronate encapsulated in liposomes that are engulfed by phagocytic cells resulting in intracellular accumulation and apoptosis [84]. Studies on intratumoural injection of oncolytic HSV-1 in mouse and rat GBM models demonstrated up to a five-fold augmented viral replication after clodronate treatment [85]. Blocking the integrin β1 receptor, expressed on the surface of macrophages, has been combined with oncolytic HSV treatment in breast cancer and glioma xenograft models. This combination decreased IFN signalling and pro-inflammatory cytokine induction in tumour cells and inhibited migration of macrophages, resulting in enhanced viral replication and cytotoxicity [86].

These findings suggested that the anti-cancer activity of OVs may specifically be impaired by macrophage-mediated responses. However, it is important to consider additional and alternative interpretations. First of all, the majority of data have been obtained using murine models, and murine in vivo studies have limited translational applications with regard to macrophages, as monocytes in mice differ from those in humans [87,88]. Furthermore, many aforementioned studies made use of athymic animal models to study the effects of immunosuppressive treatments. As a result, their negative impact on (adaptive) immune responses in oncolytic virotherapy was not exposed. In the case of immunocompetent models and administration of chemotherapeutic CPA, there might be pleiotropic effects on therapeutic efficacy. CPA has direct anti-cancer activity, and high dosages result in potent cytotoxicity and (innate) immunosuppression—whereas low dosages achieve immunostimulatory effects that include the depletion of suppressive T_REG_s and expansion of antigen-specific T-cells [79,89]. Moreover, independent of dosage, CPA exerts pro-immunogenic activities on tumour cells, inducing hallmarks of ICD [90]. This potentially enhances anti-tumour responses [64].

Thus, anti-viral immunity counteracting oncolytic virotherapy efficacy can be mediated by macrophages, but the notion that they predominantly contribute to obstructive responses could be nuanced by the choice of tumour models and pharmacological adjuvant.

### 4.2. Supportive Macrophage Responses

#### 4.2.1. Tumour Infiltration

Upon OV infection of the tumour, intrinsic anti-viral signalling activates the transcription factor NF-κB, ultimately leading to the expression of, amongst others, the chemokine CCL-2, which mediates constitutive macrophage infiltration in the TME [11]. Macrophage infiltrates are found early after OV administration [42,91]. Infection with oncolytic VV GLV-1h68 of human colorectal cancer xenografts in mice, adenovirus Delta24-RGD in a syngeneic mouse GBM model, multiple HSV-1s in orthotopic GBM xenografts, and recombinant orthopoxvirus in mouse models of colon cancer were all shown to induce an increased TAM population [43,57,92,93]. Treatment with an IL-12–encoding HSV-1 led to an enhanced influx of both activated macrophages and T-cells when compared with control OVs and improved survival in a murine glioma model [94]. The pro-inflammatory environment mediated by OVs could be potentiated by interactions with infiltrated macrophages [95,96], supporting OV therapy as substantiated by immune cell depletion studies [97]. In immunocompetent mouse models of renal adenocarcinoma and melanoma treated with the murine oncolytic adenovirus dlE102, tumour volumes were significantly reduced, and a pro-inflammatory TME was induced. They presented an increased infiltration of TAMs, NK cells, and lymphocytes, also harbouring fewer tumour-infiltrating lymphocytes expressing PD-1, a major regulator of T-cell exhaustion [98]. In recurrent GBM patients treated with oncolytic H-1 poliovirus (PV), markers of microglia and macrophage activation were detected in infected tumours [99]. In GBM patients treated with oncolytic adenovirus Delta24-RGD, cerebrospinal fluid samples showed cytokine levels indicative of an inflammatory TME, leading to further recruitment of immune cells and inflammation [100].

In summary, constitutive macrophage recruitment to the TME is regulated by the expression of CCL-2, which is induced by pro-inflammatory anti-viral signalling pathways. This macrophage infiltration has been observed in multiple in vivo tumour models treated with various OVs.

#### 4.2.2. Phenotypic Shift

Conventional therapies have been observed to further polarise macrophages towards an M2 phenotype [101]. This plasticity of resident and newly recruited macrophages in the TME could also be utilised to target and polarise TAMs towards an M1 phenotype, resulting in an anti-tumour environment [102]. Targeting TAMs through the inhibition of colony-stimulating factor-1 receptor (CSF-1R), a key macrophage signalling pathway, has been shown to counteract macrophage-mediated immune suppression by inhibiting the differentiation, proliferation and survival of M2 macrophages [103,104]. Since the presence of OVs has been demonstrated to yield an inflammatory TME, the notion was that virotherapy could be used as an inducer of M1 signalling in macrophages [105]. A phenotypic shift can be achieved by OVs through concerted action: the presence of OV particles contributes to M2 marker downregulation and soluble factors secreted from OV-infected cancer cells induce M1 marker upregulation [100]. M2-associated IRF4 and M1-associated IRF5 compete for the MYD88 complex that is also an adaptor involved in all anti-viral TLR signalling [106,107]. Through this pathway, PAMPs binding to TLRs can induce a shift in phenotype; macrophages are suggested to detect viral genetic elements by TLR9, OV-infected tumour cells by TLR2, activating IRF5 which, via the MYD88-IRF5 complex, upregulates NF-κB, IRF5, and IRF7. This ultimately inhibits M2-related signalling and leads to M1-associated gene transcription [100,108].

It has been demonstrated that tumour-resident macrophages increase in number and gain more M1 characteristics upon treatment with OVs in divergent mouse models, including oncolytic adenovirus in orthotopic GBM models [43,109] and anaplastic thyroid carcinoma xenograft models [110], as well as oncolytic HSV-1 in athymic and syngeneic models for GBM, breast cancer, and melanoma [57,97,111,112,113]. Moreover, tumour cell death mediated by the oncolytic mumps and measles paramyxoviruses was shown to be affected by monocyte-derived macrophage responses in murine breast cancer models irrespective of the polarisation state of the initial TAM population [114]. Immune cell depletion studies in murine GBM models demonstrated that these macrophages are essential for achieving therapeutic effects [97]. In clinical trials in GBM patients, Delta24-RGD, autonomous protoparvovirus H-1, and vocimagene amiretrorepvec (Toca 511) treatments were shown to induce a phenotypic shift in GBM TAMs, accompanied by pro-inflammatory cytokine secretion [100,115,116].

Recombinant OVs encoding immunomodulatory molecules provide an approach to augment this phenotypic shift. Polarisation towards M2 macrophages can be directly impaired or reversed, with an IFN-encoding [117] or soluble TGF-β receptor-encoding oncolytic viral vector. In human breast cancer bone metastases in nude mice, M2 macrophage activity and tumour progression were reduced using this approach [118]. Oncolytic virotherapy treatment of a mouse GBM model combined with an anti-PD-1 and an anti-checkpoint blockade of CTL antigen 4 (CTLA-4) immunotherapy was associated with both macrophage influx and M1-like polarisation and yielded synergistic therapeutic activity for which macrophages were required [97]; these immunostimulatory M1 phenotypes enable the induction of other adaptive immune responses.

Thus, the phenotypic spectrum of macrophages is plastic and dependent on signals from the microenvironment. TAMs have been shown to downregulate M2-associated and upregulate M1-associated markers upon OV treatment, which can be substantiated by the molecular pathways involved in anti-viral signalling and their phenotypic plasticity.

#### 4.2.3. Phagocytosis and Antigen Presentation

The OV-induced release of PAMPs, DAMPs, and cytokines into the TME promotes the maturation of APCs [119] such as M1 macrophages [57]. Maturation yields upregulated MHC-II, co-stimulatory molecules, and chemokine receptors. TAMs in a Delta24-RGD-treated GBM patient have been found to contain hexon peptides, one of the viral capsid proteins [100]. It has been demonstrated that macrophages are resistant to wild-type adenoviral replication [120], and similarly, TAMs have been shown to be non-permissive to oncolytic adenoviral replication. Thus, the observed hexon-positivity of these TAMs was presumably caused by phagocytosis. Co-culturing of in vitro polarised macrophage phenotypes with Delta24-RGD–infected tumour cells demonstrated that M1 macrophages have greater phagocytosis capacity [100], suggesting a synergistic influence of OV-induced phenotypic shift, phagocytosis, and maturation, after which digested peptides can be loaded onto MHC-II. CD169^+^ macrophages, rather than DCs, have been identified in T-cell lymphoma mouse models as being essential APCs for the induction of CD8^+^ T-cell–mediated anti-tumour immune responses [121].

The inflammatory TME mediates the recruitment and activation of T-cells [42,47,91], to which antigens can be presented either in the TME or after lymphoid migration [122,123,124]. M1 macrophages are related to inducing a T_H_1-cell response, and subsequently, activated CD8^+^ T-cells become CTLs, that have the ability to migrate to sites of tumour growth. This adaptive immune response has been associated with T-cell clones circulating and inducing lytic cell death of TAA-expressing cancer cells, both infected and uninfected [125], facilitated by the upregulation of MHC-I on cells with active NF-κB signaling. It has also been associated with an immune-mediated bystander effect, meaning that the local release of cytotoxic molecules by CTLs might induce the death of adjacent cancer cells [126]. This T-cell–mediated anti-tumour efficacy has been confirmed by studies applying cytokine-armed oncolytic HSV-1 on neuroblastoma using syngeneic models compared to athymic mice [127]. Priming of CD8^+^ T-cells can be further enhanced by antagonising tumour-induced suppression of phagocytosis with antibodies against CD47, facilitating antigen presentation by macrophages [128]. An oncolytic VV engineered to counteract anti-phagocytic CD47, replicated in tumour cells and redirected both M1 and M2 macrophages to tumour cells in vitro. Treatment of an immunocompetent osteosarcoma mouse model with this OV resulted in a significant survival advantage compared to the controls [129]. Oncolytic HSV-1 armed with an MHC-like molecule expression cassette directly stimulates macrophage-mediated antigen processing and presentation and accumulation of activated T-cells [130].

Taken together, macrophages shifted to an M1 phenotype after OV treatment can, through phagocytosis, mediate the (cross-)presentation of antigens released by ICD to elicit an adaptive immune response. This results in systemic, durable anti-tumour immunity, as observed in divergent tumour models.

#### 4.2.4. Viral Transport and Therapy Monitoring Tool

The extent and type of host anti-viral responses to OVs are primarily determined by the route of administration. Delivery preference is dependent on tumour type and OV species, but often, OVs are directly delivered through intratumoural injection [131]. Nonetheless, tumours are not always accessible, and systemic delivery may be required. Systemic administration of OVs is relatively simple, reaching multiple tumour sites, potentially treating or preventing metastases. However, this application leads to rapid recognition and elimination of the OVs by the immune system, which has been shown with oncolytic measles virus (MV) [132], Newcastle disease virus (NDV) [133], VV [134], and adenovirus [135]. Clearance could be due to humoral responses, but hepatic and splenic sequestration and absence of extravasation might also impair delivery and spread [53].

This barrier could be overcome using technologies such as liposomes and exosomes to mask the OVs and evade recognition by the immune system [136,137]. Another strategy is to mediate transport of OVs with carrier cells, which would confer both protection from neutralisation and opsonisation as well as tumour homing, without losing the biological activity of either virus or carrier cell. Various cell types have been studied as potential virus carriers, including irradiated human teratocarcinoma cells for HSV-1716 [138], neural stem or precursor cells for oncolytic HSV-1 rRp450 [139], adenovirus CRAd-S-pK7 [135,140,141] and orthopoxvirus CF33 [142], mesenchymal stem cells for oncolytic MV [143], adenovirus [144,145] and HSV [146], blood and hepatic mononuclear cells as well as lymphokine-activated (immature) DCs and NK cells for reovirus [147,148,149], and a T-cell line for Delta24-RGD [150]. They are all hypothesised to home to the TME after infection or loading ex vivo. Virus particles have been found to also naturally associate with circulating cells after systemic delivery [151]. The potential of carrier cells can vary upon their permissiveness to OV infection, natural capacity to home to tumour growth sites, ability to protect OVs from immune detection in the circulation, and transmission of OV particles to cancer cells [37]. More recently, macrophages have also been found to mediate OV transport [152]. They can be manipulated using a hypoxia-regulated construct to control oncolytic adenovirus replication. Specificity is achieved by macrophages homing to tumour-associated hypoxia upon which OV replication is activated [153]. They are also infected ex vivo [154] and reinfused. Notably, monocytes and macrophages have been found to capture circulating reovirus through antibody binding in vivo [155]. As macrophages naturally migrate to areas of tissue destruction and are actively recruited to the TME, this process contributes to the tumour targeting of systemically delivered OVs [10,156,157].

Lastly, a recent study revealed that subsets of monocytes carrying tumour- or tissue-specific proteins in their phagolysosomes could be detected in the blood of brain tumour and ischemic stroke patients [158]. These assumed recirculating tissue macrophages derived from areas of tissue damage could, in the case of virotherapy, also carry OV-derived proteins [100]. This approach may, in the future, offer a tool to monitor the presence and duration of OV replication in the tumour. Together, these findings on macrophage-mediated transport of virus particles or proteins to and from areas of tissue damage open new avenues for the delivery and monitoring of oncolytic virotherapy.

## 5. Conclusions and Future Directions

**Figure 1:**
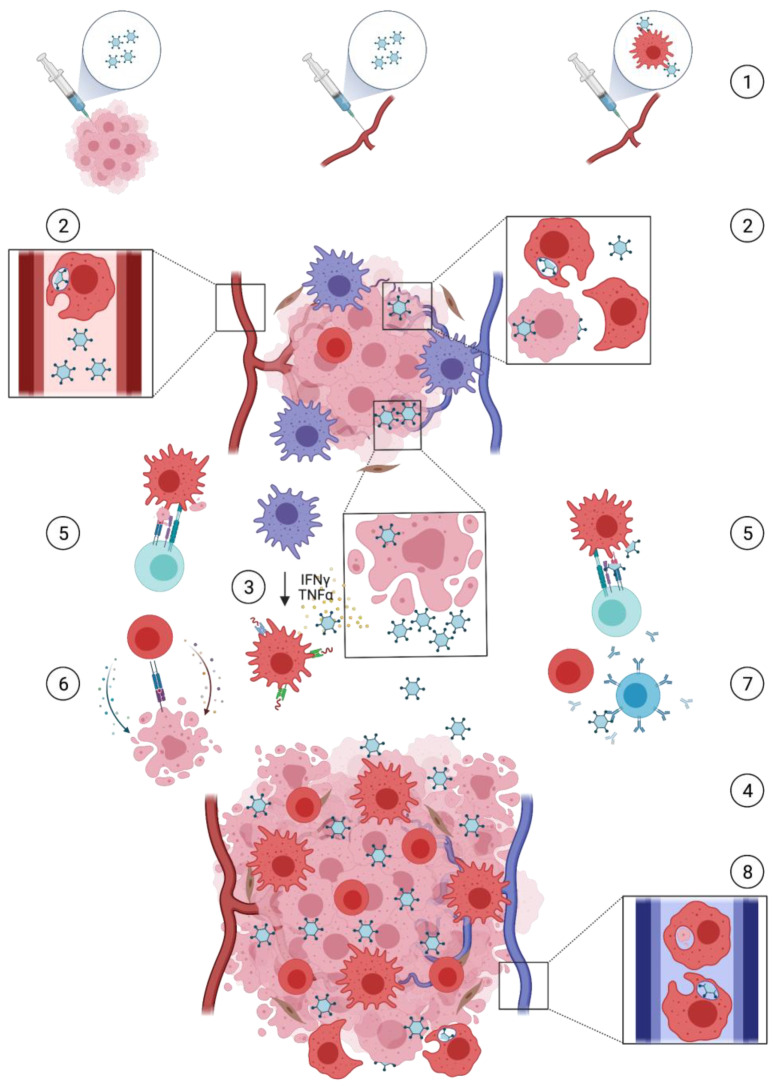
Oncolytic viruses (OVs) exert not only oncolysis, but also immune responses in which macrophages are important mediators involved in a number of key processes. Through intratumoural injection, systemic administration, or via cellular carriers (1), delivery of OVs to the immunosuppressive tumour microenvironment (TME) can be established. This is restricted by innate phagocytic activity of macrophages through clearance of virus particles and virally infected cells (2). Cancer cell infection and replication lead to immunogenic oncolysis, resulting in viral progeny spread, inflammation and antigen release. Cytokines cause a macrophage shift from a tumour-supportive M2 towards a pro-inflammatory M1 phenotype (3), sustaining inflammation. More immune cells are recruited and infiltrate the TME (4), enabling macrophage-mediated antigen presentation to T-cells (5). This adaptive immune response is multifaceted and can entail both a supportive response against tumour-associated antigens (TAAs), resulting in cytotoxic cancer cell death (6), and an obstructive response against viral antigens, facilitating T- and B-cell clearance of infected cells and virus particles (7). Macrophages may also return to the circulation carrying tumour- and virus-derived proteins and offer a tool for monitoring oncolytic virotherapy (8).

Based on current understanding, there is a multifaceted role for macrophage-mediated responses in the efficacy of oncolytic virotherapy as a cancer treatment (Figure 1).

The presence of TAMs is generally associated with a poor prognosis of human cancer patients by having an immunosuppressive influence in the TME. In oncolytic virotherapy treatment, macrophages have historically been considered to predominantly contribute to anti-viral responses, which could be nuanced and partly explained by the applied tumour models and assessed pharmacological adjuvant. At present, the insight is that macrophages also mediate virotherapy-supporting responses. They may play a crucial role in establishing adaptive anti-tumour immunity. In a variety of OV-treated tumours, macrophage infiltrates are polarised towards the pro-inflammatory, tumour-suppressive M1 phenotype. This can potentiate oncolytic virotherapy treatment through phagocytosis of (infected) cancer cells and subsequent antigen presentation, enabling a T-cell–mediated anti-tumour immune response. More recently, systemic macrophages have been found to additionally facilitate viral transport by the capturing of circulating OVs and the transmission of OV particles to tumour sites. TAMs also return to the bloodstream carrying tissue-derived proteins after completing scavenging activities in damaged tissue, which potentially enables the monitoring of OV replication in the TME. The increasing number of identified macrophage responses implies that understanding the interaction between macrophages and viruses in oncolytic virotherapy is not yet complete and is continuously evolving.

The diverse and dynamic aspects of OV infection-related immune responses have led to discordant findings on the role of macrophage-mediated responses in oncolytic virotherapy efficacy. Anti-viral and anti-tumour responses often encompass overlapping immunological pathways. However, modulating the equilibrium between these responses, aiming to maximally dampen anti-viral responses while retaining anti-tumour responses, is challenging and poorly understood but might be central to improve oncolytic virotherapy effectiveness. Specific pharmacological suppressors of the innate immune system could, for instance, be applied at low doses prior to, or at the time of, OV administration, transiently exerting their effects in interfering with anti-viral responses [129,159,160,161]. This approach is currently being investigated in a clinical phase I trial testing oncolytic HSV-1 rQNestin with single-dose CPA pre-treatment [162]. Valproic acid, an FDA-approved anti-epileptic agent with histone deacetylase inhibitory function, is also in development for treating cancer and prevents the transcriptional activity of IFN-stimulated genes [91]. Furthermore, while commonly applied clodronate liposomes non-specifically deplete macrophages, also disabling their indirect anti-cancer activity [111], a novel, more selective agent could, for instance, interfere with systemic macrophage function or characteristic phagocytosis pathways in receptor-mediated viral clearance. However, insights suggest an important role for macrophages as cellular carriers of OVs and indicate that initial restriction of systemic macrophage functions may also have unpredicted negative effects on therapeutic outcomes.

Over time, sufficient inflammation must be permitted and, when the restriction on the innate immune system has been removed, an anti-tumour response can be developed. Viral pharmacokinetics, pharmacodynamics, and kinetics of innate immune suppression in relation to the number of initial viral replication cycles require further investigation, ultimately supported by mathematical modelling of interactions and making quantitative and substantiated predictions [163]. Moreover, it needs to be elucidated whether this approach would compromise the safety profile. When optimised, this strategy could provide long-term immune-mediated anti-cancer activity.

As might be perceived, there are challenges in the broad clinical implementation of oncolytic virotherapy. In prospective preclinical studies and multi-institutional trials, it is desired – and potentially required, that (clinical) response assessments be standardised to enable comparison. To investigate the role of the innate and adaptive immune system in therapeutic efficacy, standardised analysis of immune infiltrates, circulating immune cells, intratumoural viral replication, and (degree of) ICD might be relevant. Knowing the key players during OV infection in the context of the TME will be critical for developing optimally effective oncolytic virotherapies.
